# Supplementary material for: HIVprotI: an integrated web based platform for prediction and design of HIV proteins inhibitors
Source: J Cheminform. 2018 Mar 9;10:12. doi: 10.1186/s13321-018-0266-y (PMC5845081; doi:10.1186/s13321-018-0266-y)
Supplement: Supplementary file 2 — Additional file 2. Source code of HIVProtI web server. [file 13321_2018_266_MOESM2_ESM.zip › HIVprotI_Source-code/hivprotI/weka-3-6-6/doc/weka/gui/boundaryvisualizer/package-frame.html]

weka.gui.boundaryvisualizer


weka.gui.boundaryvisualizer

Interfaces 
  
*DataGenerator*

Classes 
  
BoundaryPanel
  
BoundaryPanelDistributed
  
BoundaryVisualizer
  
KDDataGenerator
  
Messages
  
RemoteBoundaryVisualizerSubTask
  
RemoteResult
